# Roles of nitric oxide and ethyl pyruvate after peripheral nerve injury

**DOI:** 10.1186/s41232-017-0051-8

**Published:** 2017-10-02

**Authors:** Sandesh Panthi, Kripa Gautam

**Affiliations:** 1 Otago School of Biomedical Sciences, University of Otago, Otago, New Zealand; 2 China Medical University, Shenyang, People’s Republic of China

**Keywords:** Nitric oxide, Ethyl pyruvate, Peripheral nerve injury, Gasotransmitter, Wallerian degeneration

## Abstract

Short-lived reactive nitrogen species and reactive oxygen species have acquired significant attention in the field of biomedical science. Nitric oxide (NO), which was thought to be an unstable gas and pollutant, is now regarded as a gas transmitter like H_2_S and CO. NO is synthesized inside the mammalian body by l-arginine via three different isoforms of NO synthase whereas pyruvate is a glycolysis product and substrate for TCA cycle. Due to poor solubility and stability, therapeutic potential of pyruvate is limited. Ethyl pyruvate (EP) is now considered as a suitable replacement of pyruvate. In this paper, we will try to focus the effect of NO and EP in Schwann cell dedifferentiation, proliferation, nerve degeneration, and regeneration during Wallerian degeneration (WD) of peripheral nerve injury along with their neuroprotective effects, cardiovascular functioning, support in hepatic complication, etc.

## Background

Nitric oxide is a unique biological messenger which was thought to be an unstable gas and noxious pollutant for all these years, but recently, it is regarded as a fascinating gasotransmitter [1, 2]. The finding of neuromodulators like CO, NO, and H_2_S has entirely changed the prospect of synaptic transmission, and discovery of NO as a neurotransmitter has uncovered the leads for CO [3]. NO was first discovered to be a substance formed by macrophages, and they had the ability to kill tumor cells and fungi and was found to be an endothelium-derived relaxing factor [4]. All these melodramatic properties led researchers to an impression that NO is formed in the brain and finally all observations came true [5–7].

The beneficial roles of nitric oxide are changing periodically based on the research and successful discovery via several animal experiments. It was first linked with noradrenergic non-cholinergic neurotransmission in the end of the nineteenth century [8], and now it is seen as a major chemical messenger [9] and mediator of synaptic transmission and plasticity with its most appealing role in peripheral nerve injury [10, 11]. Various studies are also performed to find the role of NO in cardiovascular reflexes and vaso-neuronal coupling in CNS [12, 13]. Claimed as the molecule of the year in 1992 by Journal Science [14], the discovery of pathways and roles of NO was acknowledged by scientists with the noble prize in physiology and medicine after 6 years [15].

In addition to the physiological role of NO, this study will be mainly focused on the role of nitric oxide during Wallerian degeneration in peripheral nerves, including its role in peripheral nerve degeneration and regeneration where we will try to find its therapeutic potential in peripheral demyelination disorder.

### Synthesis and regulation of NO in nervous system

NO is biologically synthesized/produced from the amino acid l-arginine molecular oxygen as substrate via the members of NO synthase (NOS) [16]. There are three isoforms of nitric oxide synthase which are genetically different and required for NO production [17]. Three isoforms include:

1. Inducible nitric oxide synthase
2. Endothelial nitric oxide synthase
3. Neuronal nitric oxide synthase

Within the brain, NOS has been found in gamma-aminobutyric acid interneurons, aminergic, and peptidergic neurons and in neurons that use excitatory amino acid glutamate transmitter [15].

NO from nNOS and eNOS are produced in low concentration, and they act as a messenger for signal transduction while NO liberated from iNOS are in high concentration provide immunological support [18]. Under normal physiological condition, it was found that the concentration of NO fluctuates in the range of low values [19]. In the brain, during ischemia or other injuries, the level of NO rises due to production via nNOS which is triggered and modulated via glutamate [20]. On other conditions like inflammation, the level of NO, produced by iNOS, is high but temporary. So, it is often termed as a double-edged sword [21]. nNOS and eNOS are regulated via a calcium-dependent manner but iNOS via gene transcription [22]. Tissue distribution of nNOS includes CNS, PNS, skeletal muscle, and lung epithelia whereas iNOS is primarily found in macrophages, glial cells, and hepatocytes [23]. eNOS is found in endothelial cells, smooth muscle cells, and hippocampus [24].

### Nitric oxide and peripheral nerve injury

Peripheral nerve injury is a complex histopathological event often termed as Wallerian degeneration (WD) [25]. This type of injury results in hyperalgesia and allodynia which are a part of neuropathic pain. It is a well-established fact that unlike the central nervous system, peripheral nerve axons can regenerate [26]. However, due to poor axonal growth, there is a loss of sensation and muscle weakness. Some components of inflammation are helpful or sometimes harmful for nerve regeneration after peripheral nerve injury [27].

After peripheral nerve injury, a broad range of mechanisms are initiated in the proximal and distal stump of a neuron [28]. For successful growth and reinnervation of the target organ and subsequently for a successful regeneration, the following series of events occur and it is compulsory, i.e.,

1. WD including myelin breakdown and clearance of distal stump.
2. Dedifferentiation of previously myelinating Schwann cell to regeneration.

The cellular events in WD can be further reclassified into neuronal reaction, response of Schwann cell, infiltration of hematogenes cell, and their relationship with other cell of distal nerve stump [29].

And, it was found that iNOS and nNOS are both important during peripheral nerve injury, and NO released by them participates in successful WD and peripheral nerve regeneration [30, 31].

To date, few animal experiments demonstrated the effect of iNOS and nNOS in peripheral nerve degeneration and regeneration and are summarized as the critical factors in the repair of injured nerve tissues. Levy et al. used iNOS-knockout mice to observe how peripheral nerve responds in chronic constriction partial nerve injury (neuropathic pain), crush of nerve trunk, and complete nerve transection. For chronic constriction injury, they evaluated iNOS mRNA and protein expression, whereas for nerve crush and nerve transection, they used myelinated fiber morphometry and electrophysiological recording. They were able to conclude that mice lacking iNOS (iNOS-KO) has delay in the breakdown of myelinated fiber and myelinated fiber regeneration and also failed to upregulate iNOS mRNA and protein reaction. Delay in the fiber breakdown distal to injury site also delayed the whole procedure of WD of myelinated fiber. Chronic constriction model was also associated with slowed breakdown of myelinated fiber with normal initiation but dally the expression of behavioral characteristics of neuropathic pain [32, 33].

The presence of superoxide in the injury site forms peroxynitrite with NO, which is a powerful antioxidant that helps in the initiation of lipid peroxidation [34]. Lipid peroxidation assists the dissolution of myelin sheath or phagocytic effect of myelin by macrophages [35, 36]. Besides this, peripheral nerve injury is also correlated with supervision of iNOS in macrophages and Schwann cells [37]. iNOS is believed to be a contributing enzyme, and production of NO via iNOS has a role in wound healing [38], traumatic brain injury [39], MPTP-induced dopaminergic neurodegeneration [40], and cerebral ischemia [41, 42]. The level of NO is also found to be increased in the patient with Alzheimer’s disease and HIV infection [16, 32]. Another finding suggests that local non-direct inflammation of peripheral nerve after injury blocks the nerve regeneration and over production of NO via iNOS-pathway can block the repair [37]. This study used sciatic nerve transection of male SD rats as an experimental model. Using adult male wistar rats, Campuzano et al. [43] found that there are age-dependent changes in the macrophage response to injury and iNOS expression is reduced which also can affect the neurodegeneration process and injury outcome in aged individuals. Some researchers tried to find out the outcomes by disrupting the mouse iNOS gene. They carried out by deleting the promoter region of axons and disrupting some important binding domains of iNOS. In their conclusion, they stated the role of NO in septic shock and mortality [38].

**Fig. 1:**
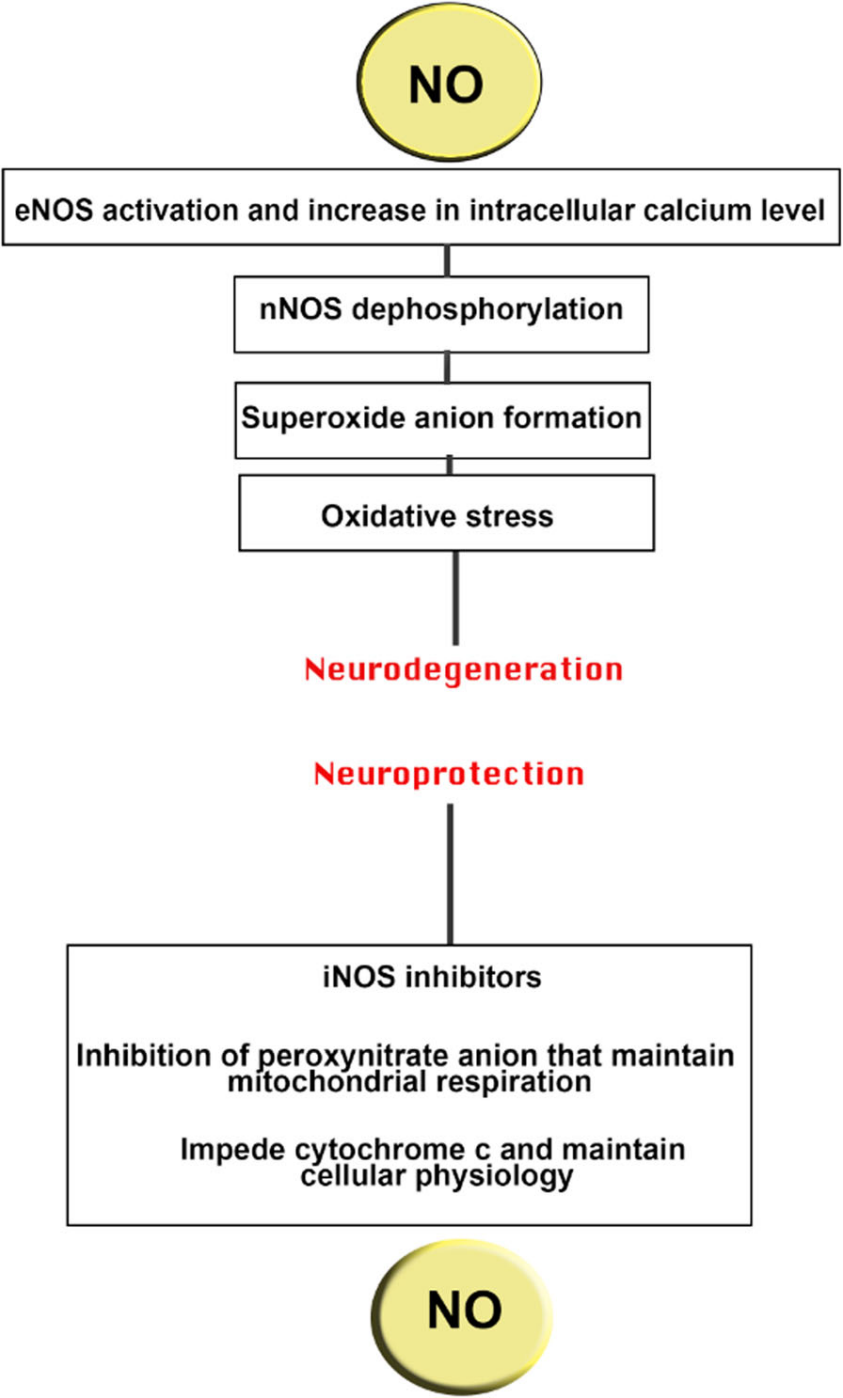
Neuroprotective and neurodegenerative aspects of NO. By activating eNOS (endothelial NO synthase), NO causes neurodegeneration which increases intracellular calcium (Ca^2+^) level following nNOS (neuronal NO synthase) dephosphorylation and oxidative stress. However, iNOS (inducible NO synthase) inhibitors inhibit peroxynitrite anion which halts cytochrome that maintains homeostasis [55]

Sciatic nerve model of mice is an ideal way for the study of effect of production of NO by three different isoforms of NOS. One study also suggests the similar type of result, where by blocking endogenous supply of NO in peripheral nerve via knocking out iNOS leads to the delay in WD which impedes the regenerative outcome [32, 43, 44]. Acetylcholinesterase histochemistry was used to examine WD. The hindered nerve regeneration in iNOS-KO mice foreground the significance of iNOS expression and liberation of NO after injury underpin the notion that effective regeneration in PNS depend on the exact timeframe of cellular and molecular degeneration procedure [18, 45, 46]. NO is also found to be connected with the removal of misdirected axons, and absence of NO or knocking out NOS also causes the disturbance in the pruning mechanism [47]. Neuroprotective and neurodegenerative roles of NO based on iNOS, eNOS, and nNOS are explained in Fig. 1.

### Ethyl pyruvate and its roles

Reactive oxygen species (ROS) scavenging property of pyruvate is well explained via various in vivo and in vitro experiments. It is found to be neuroprotective against H_2_O_2_ [48], hemorrhagic shock [49, 50], cerebral ischemic injury, zinc toxicity, pressure-induced retinal damage, and acute renal failure. It is also found to provide neuroprotective action against β-amyloid and zinc [51, 52].

A recent investigation also demonstrated the role of ethyl pyruvate in long-term neurological improvement after traumatic injury. Use of sensorimotor and cognitive neurobehavioral tests in this study suggested that ethyl pyruvate may cease the outrageous cycle of blood brain barrier disruption and damage leading to the long-term neuroprotective role. Lots of pre-clinical studies already had concluded the role of ethyl pyruvate from amelioration of hepatic neurons to acute lung injury and preservation of renal function to T-cell functions.

Recently, the effect of ethyl pyruvate in peripheral nerve injury is also identified, and it became one of the novel findings in the field of neuroscience. Park et al. [53] used 5-week-old mice to study the dedifferentiation and proliferation pattern of a Schwann cell using ethyl pyruvate during a process of WD in peripheral nerve injury. Results in this study were achieved by using antibodies against p75NGFR (p75 nerve growth factor receptor), LAMP1 (lysosomal associated membrane protein1), phosphor-p44/43MPK, and Ki67. From immunostaining and Western blot analysis, it was detected that ethyl pyruvate inhibits p-ERK1/2 expression, lysosomal activation, p75NGFR, and Ki67 expression. Sciatic explants of mice were treated with ethyl pyruvate and proved its role to inhibit Schwann cell dedifferentiation by inhibiting p-ERK1/2 and p75NGFR expression in denervated Schwann cells. It is also a well-established fact that during WD, lysosomal proteosomal protein are activated in dedifferentiated Schwann cells to remove myelin sheath debris. They remarkably observed reduced LAMP1 expression in explants treated with ethyl pyruvate. A similar type of result was discovered using cell proliferation marker Ki67 which led them to a conclusion that ethyl pyruvate also inhibits Schwann cell proliferation during WD stage of peripheral nerve injury. Effect of EP in paclitaxel-induced neuropathic pain was also investigated by researchers, but they were not able to relate mechanical allodynia of this type of neuropathic pain with EP [54].

## Conclusions

Regeneration is possible in peripheral nervous system even after nerve damage which is not possible in CNS. Peripheral nerve regeneration involves a series of events where WD is the major phenomenon. Study of these processes and effects is crucial for the treatment of demyelinating and peripheral degenerative disorders. This study tried to brief the discussion of recent findings on the importance of nitric oxide and ethyl pyruvate on peripheral nerve regeneration. Disruption of binding domains of iNOS or use of iNOS-KO mice gave the impression of delayed WD. Additionally, neurodegeneration and regeneration process is also affected by the age of an individual. The role of NO is also found in removal of misdirected axons and enhance the pruning mechanism of axons. Importance and role of ethyl pyruvate in and after peripheral nerve injury are still in the infant stage of finding. However, recently, few researchers claimed that ethyl pyruvate inhibits Schwann cell dedifferentiation and proliferation. It is also believed that reduction of oxidative stress is the result of this inhibition. Thus, for the treatment of disorders related to peripheral nerve injury, new strategy and finding will be helpful.

## Abbreviations

CNS: Central nervous system
CO: Carbon-monoxide
eNOS: Endothelial nitric oxide synthase
EP: Ethyl pyruvate
H_2_O_2_: Hydrogen peroxide
H_2_S: Hydrogen sulfide
HIV: Human immuno virus
iNOS: Inducible nitric oxide synthase
LAMP1: Lysosomal-associated membrane protein
MPK: Mitogen-activated protein kinase
mRNA: Messenger ribonucleic acid
nNOS: Neuronal nitric oxide synthase
NO: Nitric oxide
NOS: Nitric oxide synthase
p75NGFR: p75 nerve growth factor receptor
p-ERK1/2: p-extracellular regulated kinase 1/2
PNS: Peripheral nervous system
ROS: Reactive oxygen species
TCA: Tri-carboxylic acid
WD: Wallerian degeneration

## References

[1] Toledo JC, Augusto O (2012). Connecting the chemical and biological properties of nitric oxide. Chem Res Toxicol.

[2] Basudhar D, Ridnour LA, Cheng R, Kesarwala AH, Heinecke J, Wink DA (2016). Biological signaling by small inorganic molecules. Coord Chem Rev.

[3] Panthi S, Chung H-J, Jung J, Jeong NY (2016). Physiological importance of hydrogen sulfide: emerging potent neuroprotector and neuromodulator. Oxidative Medicine and Cellular Longevity 2016.

[4] Loscalzo J (2013). The identification of nitric oxide as endothelium-derived relaxing factor. Circ Res.

[5] Faro MLL, Fox B, Whatmore JL, Winyard PG, Whiteman M (2014). Hydrogen sulfide and nitric oxide interactions in inflammation. Nitric Oxide.

[6] Ersoy A, Koc ER, Sahin S, Duzgun U, Acar B, Ilhan A (2014). Possible effects of rosuvastatin on noise-induced oxidative stress in rat brain. Noise Health.

[7] Garry P, Ezra M, Rowland M, Westbrook J, Pattinson K (2015). The role of the nitric oxide pathway in brain injury and its treatment—from bench to bedside. Exp Neurol.

[8] Rajfer J, Aronson WJ, Bush PA, Dorey FJ, Ignarro LJ (1992). Nitric oxide as a mediator of relaxation of the corpus cavernosum in response to nonadrenergic, noncholinergic neurotransmission. N Engl J Med.

[9] Lamattina L, García-Mata C, Graziano M, Pagnussat G (2003). Nitric oxide: the versatility of an extensive signal molecule. Annu Rev Plant Biol.

[10] Zochodne D, Levy D (2005). Nitric oxide in damage, disease and repair of the peripheral nervous system. Cell Mole Biol (Noisy-le-Grand, France).

[11] Tanabe M, Nagatani Y, Saitoh K, Takasu K, Ono H (2009). Pharmacological assessments of nitric oxide synthase isoforms and downstream diversity of NO signaling in the maintenance of thermal and mechanical hypersensitivity after peripheral nerve injury in mice. Neuropharmacology.

[12] Zanzinger J (1999). Role of nitric oxide in the neural control of cardiovascular function. Cardiovasc Res.

[13] Gkaliagkousi E, Ferro A (2010). Nitric oxide signalling in the regulation of cardiovascular and platelet function. Front Biosci (Landmark edition).

[14] Cech TR, Bennett D, Jasny B, Kelner KL, Miller LJ, Szuromi PD, Voss DF, Kiberstis PA, Parks S, Ray LB (1992). The molecule of the year. Science.

[15] Zhou L, Zhu D-Y (2009). Neuronal nitric oxide synthase: structure, subcellular localization, regulation, and clinical implications. Nitric Oxide.

[16] Calabrese V, Mancuso C, Calvani M, Rizzarelli E, Butterfield DA, Stella AMG (2007). Nitric oxide in the central nervous system: neuroprotection versus neurotoxicity. Nat Rev Neurosci.

[17] Boissel J-P, Schwarz PM, Förstermann U (1998). Neuronal-type NO synthase: transcript diversity and expressional regulation. Nitric Oxide.

[18] Prast H, Philippu A (2001). Nitric oxide as modulator of neuronal function. Prog Neurobiol.

[19] Tieu K, Ischiropoulos H, Przedborski S (2003). Nitric oxide and reactive oxygen species in Parkinson’s disease. IUBMB Life.

[20] Keynes RG, Garthwaite J (2004). Nitric oxide and its role in ischaemic brain injury. Curr Mol Med.

[21] Mocellin S, Bronte V, Nitti D (2007). Nitric oxide, a double edged sword in cancer biology: searching for therapeutic opportunities. Med Res Rev.

[22] Kim Y, Moon JS, Lee KS, Park SY, Cheong J, Kang HS, Lee HY, Do Kim H (2004). Ca 2+/calmodulin-dependent protein phosphatase calcineurin mediates the expression of iNOS through IKK and NF-κB activity in LPS-stimulated mouse peritoneal macrophages and RAW 264.7 cells. Biochem Biophys Res Commun.

[23] Förstermann U, Sessa WC (2012). Nitric oxide synthases: regulation and function. Eur Heart J.

[24] Albrecht EW, Stegeman CA, Heeringa P, Henning RH, van Goor H (2003). Protective role of endothelial nitric oxide synthase. J Pathol.

[25] Dubový P (2011). Wallerian degeneration and peripheral nerve conditions for both axonal regeneration and neuropathic pain induction. Ann Anat Anat Anz.

[26] Gaudet AD, Popovich PG, Ramer MS (2011). Wallerian degeneration: gaining perspective on inflammatory events after peripheral nerve injury. J Neuroinflammation.

[27] Coleman MP, Freeman MR (2010). Wallerian degeneration, wlds, and nmnat. Annu Rev Neurosci.

[28] Burnett MG, Zager EL (2004). Pathophysiology of peripheral nerve injury: a brief review. Neurosurg Focus.

[29] Mcdonald D, Cheng C, Chen Y, Zochodne D (2006). Early events of peripheral nerve regeneration. Neuron Glia Biol.

[30] Martucci C, Trovato AE, Costa B, Borsani E, Franchi S, Magnaghi V, Panerai AE, Rodella LF, Valsecchi AE, Sacerdote P (2008). The purinergic antagonist PPADS reduces pain related behaviours and interleukin-1β, interleukin-6, iNOS and nNOS overproduction in central and peripheral nervous system after peripheral neuropathy in mice. Pain.

[31] Guan Y, Yaster M, Raja SN, Tao Y-X (2007). Genetic knockout and pharmacologic inhibition of neuronal nitric oxide synthase attenuate nerve injury-induced mechanical hypersensitivity in mice. Mol Pain.

[32] Levy D, Kubes P, Zochodne DW (2001). Delayed peripheral nerve degeneration, regeneration, and pain in mice lacking inducible nitric oxide synthase. J Neuropathol Exp Neurol.

[33] Levy D, Höke A, Zochodne DW (1999). Local expression of inducible nitric oxide synthase in an animal model of neuropathic pain. Neurosci Lett.

[34] Hogg N, Kalyanaraman B (1999). Nitric oxide and lipid peroxidation. Biochim Biophys Acta Bioenerg.

[35] Rubbo H, Radi R, Trujillo M, Telleri R, Kalyanaraman B, Barnes S, Kirk M, Freeman BA (1994). Nitric oxide regulation of superoxide and peroxynitrite-dependent lipid peroxidation. Formation of novel nitrogen-containing oxidized lipid derivatives. J Biol Chem.

[36] Radi R, Beckman JS, Bush KM, Freeman BA (1991). Peroxynitrite-induced membrane lipid peroxidation: the cytotoxic potential of superoxide and nitric oxide. Arch Biochem Biophys.

[37] McDonald DS, Cheng C, Martinez JA, Zochodne DW (2007). Regenerative arrest of inflamed peripheral nerves: role of nitric oxide. Neuroreport.

[38] Yamasaki K, Edington HD, McClosky C, Tzeng E, Lizonova A, Kovesdi I, Steed DL, Billiar TR (1998). Reversal of impaired wound repair in iNOS-deficient mice by topical adenoviral-mediated iNOS gene transfer. J Clin Investig.

[39] Sinz EH, Kochanek PM, Dixon CE, Clark RS, Carcillo JA, Schiding JK, Chen M, Wisniewski SR, Carlos TM, Williams D (1999). Inducible nitric oxide synthase is an endogenous neuroprotectant after traumatic brain injury in rats and mice. J Clin Invest.

[40] Liberatore GT, Jackson-Lewis V, Vukosavic S, Mandir AS, Vila M, McAuliffe WG, Dawson VL, Dawson TM, Przedborski S (1999). Inducible nitric oxide synthase stimulates dopaminergic neurodegeneration in the MPTP model of Parkinson disease. Nat Med.

[41] Nogawa S, Forster C, Zhang F, Nagayama M, Ross ME, Iadecola C (1998). Interaction between inducible nitric oxide synthase and cyclooxygenase-2 after cerebral ischemia. Proc Natl Acad Sci.

[42] Iadecola C, Zhang F, Xu S, Casey R, Ross ME (1995). Inducible nitric oxide synthase gene expression in brain following cerebral ischemia. J Cereb Blood Flow Metab.

[43] Campuzano O, Castillo-Ruiz M, Acarin L, Castellano B, Gonzalez B (2008). Distinct pattern of microglial response, cyclooxygenase-2, and inducible nitric oxide synthase expression in the aged rat brain after excitotoxic damage. J Neurosci Res.

[44] Keilhoff G, Fansa H, Wolf G (2002). Differences in peripheral nerve degeneration/regeneration between wild type and neuronal nitric oxide synthase knockout mice. J Neurosci Res.

[45] Turner JE, Glaze KA (1977). The early stages of Wallerian degeneration in the severed optic nerve of the newt (Triturus viridescens). Anat Rec.

[46] Yuan Q, Su H, Chiu K, Lin Z-X, Wu W (2014). Assessment of the rate of spinal motor axon regeneration by choline acetyltransferase immunohistochemistry following sciatic nerve crush injury in mice: laboratory investigation. J Neurosurg.

[47] Rabinovich D, Yaniv SP, Alyagor I, Schuldiner O (2016). Nitric oxide as a switching mechanism between axon degeneration and regrowth during developmental remodeling. Cell.

[48] Wang X, Perez E, Liu R, Yan L-J, Mallet RT, Yang S-H (2007). Pyruvate protects mitochondria from oxidative stress in human neuroblastoma SK-N-SH cells. Brain Res.

[49] Mongan PD, Capacchione J, West S, Karaian J, Dubois D, Keneally R, Sharma P (2002). Pyruvate improves redox status and decreases indicators of hepatic apoptosis during hemorrhagic shock in swine. Am J Phys Heart Circ Phys.

[50] Mongan PD, Capacchione J, Fontana JL, West S, Bunger R (2001). Pyruvate improves cerebral metabolism during hemorrhagic shock. Am J Phys Heart Circ Phys.

[51] Fink M (2007). Ethyl pyruvate: a novel anti-inflammatory agent. J Intern Med.

[52] Kawahara M, Kato-Negishi M, Kuroda Y (2002). Rapid communication: pyruvate blocks zinc-induced neurotoxicity in immortalized hypothalamic neurons. Cell Mol Neurobiol.

[53] Park BS (2015). A novel effect of ethyl pyruvate in Schwann cell de differentiation and proliferation during Wallerian degeneration. Anim Cells Syst.

[54] Choi SS (2013). Effect of ethyl pyruvate on Paclitaxel-induced neuropathic pain in rats. Kor J Pain.

[55] Shefa U, Geun Yeo S, Kim MS (2017). Role of gasotransmitters in oxidative stresses, neuroinflammation, and neuronal repair. Biomed Res Int.

