# Correction to: Roles of nitric oxide and ethyl pyruvate after peripheral nerve injury

**DOI:** 10.1186/s41232-019-0091-3

**Published:** 2019-01-23

**Authors:** Sandesh Panthi, Kripa Gautam, Junyang Jung

**Affiliations:** 1 Otago School of Biomedical Sciences, University of Otago, Otago, New Zealand; 2 China Medical University, Shenyang, People’s Republic of China; 3 Department of Anatomy and Neurobiology, College of Medicine, Kyung Hee University, Seoul, Korea

**Correction to: Inflamm Regen (2017) 37:20**

10.1186/s41232-017-0051-8

After publication of the original article [1], it came to our attention that Professor Junyang Jung was omitted from the authorship. The correct authorship is Sandesh Panthi, Kripa Gautam, and Junyang Jung.

In addition, the following funding information should have been included as part of Declarations:

“Sandesh Panthi was supported by Basic Science Research Program through the Korean National Research Foundation (NRF) funded by the Ministry of Science, ICT and Future Planning (Professor Junyang Jung, 2015R1A2A2A01002735).”
